# Peptide-Based TNF-α-Binding Decoy Therapy Mitigates Lipopolysaccharide-Induced Liver Injury in Mice

**DOI:** 10.3390/ph13100280

**Published:** 2020-09-29

**Authors:** Chao-Yuan Chang, Hao-Jen Hsu, Jossen Foo, Hung-Jen Shih, Chun-Jen Huang

**Affiliations:** 1 Department of Anesthesiology, Wan Fang Hospital, Taipei Medical University, Taipei 116, Taiwan; yuanc669@tmu.edu.tw (C.-Y.C.); 105271@w.tmu.edu.tw (J.F.); 2 Integrative Research Center for Critical Care, Wan Fang Hospital, Taipei Medical University, Taipei 116, Taiwan; 3 Graduate Institute of Clinical Medicine, College of Medicine, Taipei Medical University, Taipei 110, Taiwan; 4 Department of Life Sciences, College of Medicine, Tzu Chi University, Hualien 970, Taiwan; hjhsu32@mail.tcu.edu.tw; 5 Department of Urology, Wan Fang Hospital, Taipei Medical University, Taipei 116, Taiwan; 6 Department of Urology, School of Medicine, College of Medicine, Taipei Medical University, Taipei 110, Taiwan; 7 Department of Anesthesiology, School of Medicine, College of Medicine, Taipei Medical University, Taipei 110, Taiwan

**Keywords:** TNF-α, necroptosis, pyroptosis, apoptosis, autophagy, mitochondrial injury

## Abstract

A peptide named SEM18, possessing structural similarity to the binding site of tumor necrosis factor (TNF)-α to TNF receptor 1 (TNFR1), was designed. We investigated whether the SEM18 peptide can mitigate lipopolysaccharide (LPS)-induced liver injury in mice. Adult male Balb/cJ mice received LPS (15 mg/kg; LPS group) or LPS plus SEM18 (LSEM group). Control groups were run simultaneously. At 2 h after LPS, the first dose of SEM18 (0.3 mg/kg) was administered, followed by three supplemental doses of SEM18 (0.15 mg/kg, every 2 h). At 24 h after LPS, surviving mice were euthanized for analyses. Compared with the LPS group, binding of TNF-α to TNFR1 in liver tissues was significantly lower in the LSEM group (*p* < 0.001). Plasma concentrations of aspartate transaminase and alanine transaminase, as well as Suzuki’s scores (liver damage assessment), wet/dry weight ratios, levels of polymorphonuclear neutrophil infiltration, and levels of mitochondrial injury in liver tissues, of the LSEM group were significantly lower than in the LPS group (all *p* < 0.05). Levels of necroptosis, pyroptosis, apoptosis, and autophagy upregulation in liver tissues in the LSEM group were also significantly lower than in the LPS group (all *p* < 0.05). Notably, exogenous TNF-α counteracted these effects of SEM18. SEM18 peptide mitigates LPS-induced liver injury in mice.

## 1. Introduction

Tumor necrosis factor-α (TNF-α), a 17 kDa protein and a pleiotropic cytokine, is derived mainly from infectious stimulus-activated immune cells (e.g., macrophages) via a Toll-like receptor (TLR)/nuclear factor-κB (NF-κB) pathway during sepsis [1]. In the early phase of sepsis, TNF-α is synthesized and released into systemic circulation [2]. This early surge of circulating TNF-α binds to its corresponding TNF receptor 1 (TNFR1, a specific transmembrane death receptor) to activate immune cells (i.e., macrophages) [2,3] and enhance the release of inflammatory mediators, subsequently leading to the development of a systemic inflammatory response during sepsis [2,3].

Upon binding with TNFR1, TNF-α can directly trigger the cell death processes of necroptosis and apoptosis via the death receptor-dependent pathways and cause cell death and damage to vital organs (e.g., liver) [4,5,6]. Of note, necroptosis can also activate the nucleotide-binding oligomerization domain-like receptor protein 3 (NLRP3) inflammasome and subsequently trigger pyroptosis [7,8,9]. In addition, TNF-α together with nitric oxide from inducible nitric oxide synthase can cause mitochondrial injury and dysfunction and subsequently induce apoptosis (i.e., the mitochondrial-dependent pathway) [10,11]. Mitochondrial dysfunction can also trigger the processes of necroptosis and pyroptosis [12,13]. Moreover, autophagy is a process of self-degradation, and crosstalk between autophagy and various processes of cell death has been highlighted [14]. Collectively, these data demonstrated that organ injuries mediated by TNF-α and the TNF-α/TNFR1 pathway during sepsis may involve mitochondrial injury/dysfunction and upregulation of the cell death processes of necroptosis, pyroptosis, apoptosis, and autophagy. These data further highlighted that TNF-α and TNFR1 can be novel therapeutic targets against sepsis.

In line with this concept, several drugs and biologics have been developed, including monoclonal anti-TNF-α antibodies (e.g., infliximab, golimumab, and adalimumab) and immunoglobulin G that can bind and inactivate TNF-α (e.g., etanercept) [15,16]. Clinical data have revealed that these TNF-α targeting agents may exert certain beneficial effects against sepsis (e.g., improving survival in patients with clinical sepsis) [17]. However, one main concern regarding the current TNF-α-targeting agents is that these agents are expensive [16]. Moreover, the current TNF-α-targeting agents are all large molecules, which carry the potential threat of causing significant side effects [16].

Peptides are highly selective, efficacious, relatively safe, and well tolerated [18]. Using the molecular docking simulation technique, the coauthor Hsu designed the SEM18 peptide possessing a structur similar to the TNFR1-binding site of TNF-α [19,20,21]. The equilibrium Kd (i.e., the equilibrium dissociation constant) for the binding of SEM18 peptide to TNF-α was measured to be 68.3 μM [21]. In vitro data confirmed that the SEM18 peptide can serve as a TNF-α-binding decoy to significantly inhibit TNF-α binding to TNFR1 [21]. As peptides are not as expensive, we conjectured that the SEM18 peptide can be an attractive alternative to current TNF-α-targeting agents if its therapeutic effects are confirmed.

To further elucidate this issue, we conducted this study with the hypothesis that the SEM18 peptide can mitigate endotoxin-induced organ injuries in septic mice. As the liver is vulnerable during sepsis [4,5,6], we decided to focus on elucidating liver injury in this study. Effects of the SEM18 peptide on modulating mitochondrial injury, necroptosis, pyroptosis, apoptosis, and autophagy in liver tissues, as well as systemic inflammatory responses and survival, in experimental septic mice were also investigated.

## 2. Results

### 2.1. Pharmacokinetic Analysis and Tissue Distribution of SEM18 Peptide

**Figure 1:**
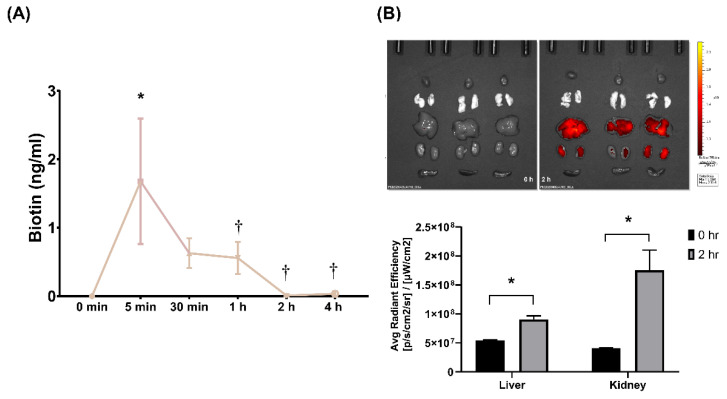
Pharmacokinetics and biodistribution of SEM18 peptide. (**A**) Pharmacokinetic analysis of SEM18 peptide. A single dose of biotin-conjugated SEM18 peptide (0.3 mg/kg) was administered. The plasma concentrations of SEM18 peptide were determined via measuring plasma biotin concentrations using enzyme-linked immunosorbent assay (ELISA), as measured at 0 min (baseline), 5 min, 30 min, 1 h, 2 h, and 4 h after intraperitoneal (i.p.) administration of SEM18 peptide; (**B**) Biodistribution of SEM18 peptide, as measured at 0 h (baseline) and 2 h after i.p. administration using ex vivo bioluminescence imaging assay. Data are presented as the mean ± standard deviation. Data of pharmacokinetics and biodistribution were derived from three mice at each time point. * *p* < 0.05 vs. the baseline value; ^†^ *p* < 0.05 vs. the value measured at 5 min after administration.

Pharmacokinetic analysis of SEM18 peptide was illustrated in Figure 1A. A single dose of biotin-conjugated SEM18 peptide (0.3 mg/kg) was administered intraperitoneally (i.p.). The plasma concentrations of SEM18 peptide were determined via measuring biotin concentrations in plasma using enzyme-linked immunosorbent assay (ELISA). Our data demonstrated that the plasma concentration of SEM18 peptide measured at 5 min after administration was significantly higher than the baseline value (i.e., 0 min, *p* = 0.002). The plasma concentration of SEM18 peptide measured at 5 min was higher than that measured at 30 min after administration, whereas the difference did not reach statistical significance. Notably, the plasma concentration of SEM18 peptide measured at 5 min was significantly higher than that measured at 1 h after administration (*p* = 0.042). Moreover, the plasma concentrations of SEM18 peptide measured at 2 and 4 h after administration were almost undetectable, and both were significantly lower than that measured at 5 min after administration (both *p* = 0.003). These data indicated that the in vivo half-life of the SEM18 peptide was estimated to be approximately 60 min with i.p. administration.

The biodistribution of the SEM18 peptide is illustrated in Figure 1B. A single dose of rhodamine B-conjugated SEM18 peptide (0.3 mg/kg, i.p.) was administered. Biodistribution of the SEM18 peptide was measured at 0 h (baseline) and 2 h after SEM18 peptide administration using a bioluminescence image assay. Our data demonstrated that the liver and kidney, but not the heart, lungs, and spleen, had significant fluorescence signals of the SEM18 peptide. Analysis further revealed that the fluorescence signal intensities of liver and kidney measured at 2 h after SEM18 peptide administration were both significantly higher than the baseline values (both *p* < 0.001). These data confirmed significant SEM18 peptide distribution in liver and kidney tissues with i.p. administration.

### 2.2. SEM18 Peptide Increases Survival and Decreases Plasma Cytokine Concentrations in Lipopolysaccharide (LPS)-Treated Mice

LPS (*Escherichia coli* endotoxin) was used to induce monomicrobial sepsis and liver injury. Adult male mice were randomly allocated to receive i.p. injections of LPS (15 mg/kg), LPS plus SEM18 peptide, or LPS plus the control peptide (CP) (denoted as the LPS, LSEM, and LCP groups, respectively). For the respective controls of the LPS-treated groups, another set of mice were randomly allocated to receive i.p. injections of normal saline (NS; 0.5 mL), NS plus SEM18 peptide, or NS plus CP (denoted as the sham, SEM, and CP groups, respectively). At 2 h after LPS or NS, the first dose of the peptide (SEM18 or CP, 0.3 mg/kg, i.p.) was administered, followed by three supplemental doses of the peptide (SEM18 or CP, 0.15 mg/kg, every 2 h, i.p.).

**Figure 2:**
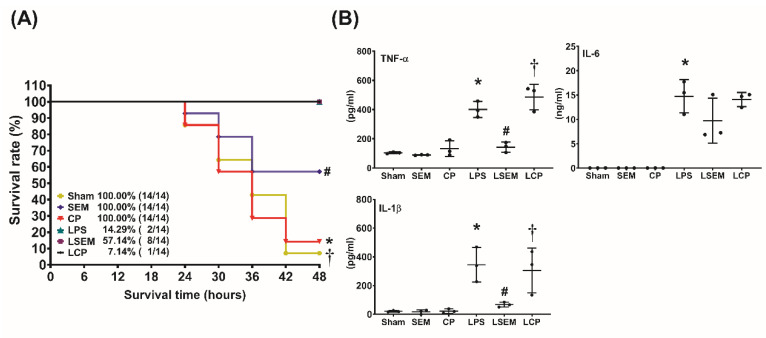
SEM18 peptide increases survival and decreases systemic inflammatory responses in endotoxin-treated mice. (**A**) The 48 h survival rate, as measured at 48 h after lipopolysaccharide (LPS) or normal saline (NS) administration. (**B**) Plasma concentrations of tumor necrosis factor (TNF)-α, interleukin-1β (IL-1β), and IL-6, as measured at 24 h after LPS or NS administration using enzyme-linked immunosorbent assay (ELISA). Sham: the NS group; SEM: the NS plus SEM18 peptide group. CP: the NS plus control peptide (CP) group; LPS: the LPS (15 mg/kg) group; LSEM: the LPS (15 mg/kg) plus SEM18 peptide group; LCP: the LPS (15 mg/kg) plus CP group. Data are presented as the mean ± standard deviation. Data of survival rates and ELISA were derived from 14 and three mice from each group, respectively. * *p* < 0.05, the LPS group vs. the sham group; ^#^ *p* < 0.05, the LSEM group vs. the LPS group; ^†^ *p* < 0.05, the LCP group vs. the LSEM group.

The 48 h survival rates are illustrated in Figure 2A. The 48-h survival rates were measured at 48 h after LPS or NS administration. The 48 h survival rates in the sham, SEM18, and CP groups were all 100%. The 48 h survival rate in the LPS group (14.3%) was significantly lower than that in the sham group (*p* < 0.001). By contrast, the 48 h survival rate in the LSEM group (57.1%) was significantly higher than those in the LPS and the LCP groups (7.1%) (*p* = 0.030 and 0.026, respectively).

Figure 2B illustrates the plasma concentrations of inflammatory cytokines, including TNF-α, interleukin (IL)-1β, and IL-6, measured at 24 h after LPS or NS administration using ELISA. The plasma TNF-α, IL-1β, and IL-6 concentrations were low in the sham, SEM, and CP groups, whereas the plasma concentrations of TNF-α, IL-1β, and IL-6 in the LPS group were significantly higher than those in the sham group (all *p* < 0.001). Moreover, the plasma concentrations of TNF-α and IL-1β in the LSEM group were significantly lower than those in the LPS and LCP groups (all *p* < 0.001).

Of note, the expression level of plasma IL-6 in the LPS group was higher than that in the LSEM group. However, the between-group differences did not reach statistical significance. Our analysis revealed that the plasma IL-6 concentrations in the LPS, LSEM, and LCP groups were not significantly different.

### 2.3. SEM18 Peptide Inhibits LPS-Induced Binding of TNF-α to TNFR1 in the Liver

**Figure 3:**
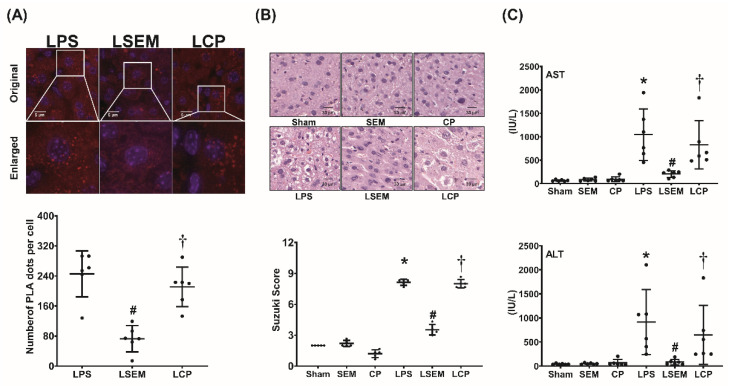
SEM18 peptide mitigates tumor necrosis factor (TNF)-α binding to TNF receptor 1 (TNFR1) and decreases liver injury induced by endotoxin. (**A**) Binding of TNF-α to TNFR1 in liver tissues, as measured at 24 h after lipopolysaccharide (LPS) or normal saline (NS) administration using immunofluorescence staining of proximity ligation assay (PLA). Red dots: positive PLA signal showing protein–protein interactions between TNF-α and TNFR1 in liver tissues. Blue dots: DAPI (4′,6-diamidino-2-phenylindole) stain showing a cell nucleus in liver tissues. (**B**) Representative microscopic findings of the liver tissues stained with hematoxylin–eosin (200×) and the organ injury scores (Suzuki’s scores), as measured at 24 h after LPS or NS administration. (**C**) Plasma concentrations of aspartate aminotransferase (AST) and alanine aminotransferase (ALT), as measured at 24 h after LPS or NS administration. Sham: the NS group; SEM: the NS plus SEM18 peptide group; CP: the NS plus control peptide (CP) group; LPS: the LPS (15 mg/kg) group; LSEM: the LPS (15 mg/kg) plus SEM18 peptide group; LCP: the LPS (15 mg/kg) plus CP group. Data are presented as the mean ± standard deviation. Data related to PLA, microscopic findings, Suzuki’s score, AST, and ALT were derived from five, five, six, and six mice from each group, respectively. * *p* < 0.05, the LPS group vs. the sham group; ^#^ *p* < 0.05, the LSEM group vs. the LPS group; ^†^ *p* < 0.05, the LCP group vs. the LSEM group.

The binding assay of TNF-α to TNFR1 in the liver measured at 24 h after LPS or NS administration using a proximity ligation assay (PLA) is illustrated in Figure 3A. Our data demonstrated that hepatic PLA signals in the sham, SEM, and CP groups were undetected (data not shown). Notably, PLA signal intensity was significantly higher in the LPS group than in the sham group (*p* < 0.001). Moreover, PLA signal intensity in the LSEM group was significantly lower than that in the LPS and LCP groups (both *p* < 0.001).

### 2.4. SEM18 Peptide Mitigates LPS-Induced Liver Injury

Histological analysis and Suzuki’s scores (liver damage assessment) of the liver tissues measured at 24 h after LPS or NS administration are illustrated in Figure 3B. Figure 3C illustrates the plasma concentrations of aspartate transaminase (AST) and alanine transaminase (ALT) measured at 24 h after LPS or NS administration. Histological analysis of the sham, SEM, and CP groups revealed no significant liver injury, and the Suzuki’s scores and plasma AST and ALT concentrations were low. In contrast, significant liver injury in the LPS group was observed. The Suzuki’s scores and plasma AST and ALT concentrations in the LPS group were significantly higher than in the sham group (all *p* < 0.001). Histological analysis further revealed lower levels of liver injury in the LSEM group than in the LPS group. The Suzuki’s scores and plasma AST and ALT concentrations in the LSEM group were significantly lower than in the LPS group (*p* < 0.001, *p* < 0.001, and *p* = 0.017, respectively). Moreover, analyses revealed that the differences between the LSEM and LCP groups paralleled those between the LSEM and LPS groups.

### 2.5. SEM18 Peptide Mitigates LPS-Induced Inflammation and Mitochondrial Injury in the Liver

**Figure 4:**
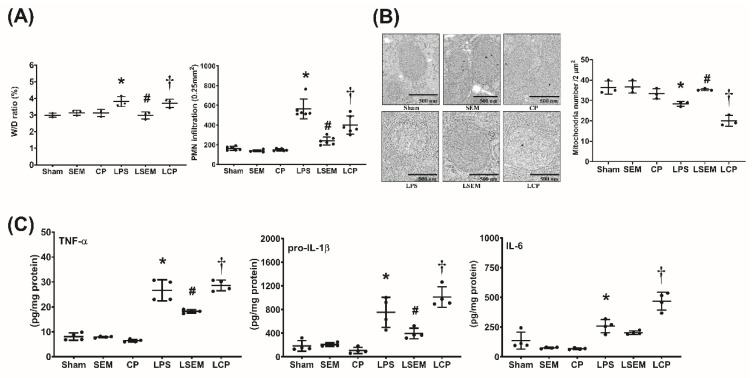
SEM18 peptide mitigates endotoxin-induced inflammation and mitochondrial injury in liver tissues induced by endotoxin. (**A**) Wet/dry (W/D) weight ratio and polymorphonuclear leukocyte (PMN) infiltration levels in liver tissues, as measured at 24 h after lipopolysaccharide (LPS) or normal saline (NS) administration. (**B**) Hepatic concentrations of tumor necrosis factor-α (TNF-α), pro-interleukin-1β (pro-IL-1β), and IL-6, as measured at 24 h after LPS or NS administration using enzyme-linked immunosorbent assay (ELISA). (**C**) Representative transmission electron microscopic images of mitochondria in liver tissues (12,000×) and the mean intact mitochondrion numbers (per 2 μm^2^) in liver tissues, as measured at 24 h after LPS or NS administration. Sham: the NS group; SEM: the NS plus SEM18 peptide group; CP: the NS plus control peptide (CP) group; LPS: the LPS (15 mg/kg) group; LSEM: the LPS (15 mg/kg) plus SEM18 peptide group; LCP: the LPS (15 mg/kg) plus C group. Data are the mean ± standard deviation. Data of W/D weight ratio, PMN infiltration, ELISA, and mitochondrial injury assay were derived from three, six, four, and three mice from each group, respectively. * *p* < 0.05, the LPS group vs. the sham group; ^#^ *p* < 0.05, the LSEM group vs. the LPS group; ^†^ *p* < 0.05, the LCP group vs. the LSEM group.

Analyses of wet/dry weight ratio (i.e., liver water content) and polymorphonuclear neutrophil (PMN) infiltration in liver tissues measured a 24 h after LPS or NS administration are illustrated in Figure 4A. Figure 4B,C illustrate hepatic concentrations of inflammatory cytokines and mitochondrial injury in liver tissues measured at 24 h after LPS or NS administration. Analyses of wet/dry weight ratio, PMN infiltration, inflammatory cytokine concentrations, and mitochondrial injury in the liver revealed similar pictures in the sham, SEM, and CP groups. Notably, the wet/dry weight ratio and level of PMN infiltration in liver tissues of the LPS group were significantly higher than in the sham group (*p* = 0.006 and *p* < 0.001, respectively). The hepatic concentrations of TNF-α, pro-IL-1β, and IL-6 in the LPS group were significantly higher than those in the sham group (all *p* < 0.001). On the contrary, the mean intact mitochondrion number in liver tissues of the LPS group was significantly lower than that in the sham group (*p* = 0.015). The wet/dry weight ratio and PMN infiltration level in the LSEM group were significantly lower than those in the LPS group (*p* = 0.005 and *p* = 0.014, respectively). The hepatic concentrations of TNF-α and pro-IL-1β in the LSEM group were significantly lower than those in the LPS group (*p* < 0.001 and *p* = 0.019, respectively). Of note, the hepatic IL-6 concentration in the LPS group was higher than that in the LSEM group. However, the between-group differences did not reach statistical significance. Therefore, hepatic IL-6 concentrations in the LSEM and LPS groups were not significantly different (Figure 3C). Notably, the mean intact mitochondrion number in the LSEM group was significantly higher than that in the LPS group (*p* = 0.035).

Moreover, analyses also revealed that the differences between the LSEM and LCP groups paralleled those between the LSEM and LPS groups, except that the hepatic IL-6 concentration in the LCP group was significantly higher than that in the LSEM group (*p* < 0.001).

### 2.6. SEM18 Peptide Mitigates LPS-Induced Necroptosis and Pyroptosis in the Liver

**Figure 5:**
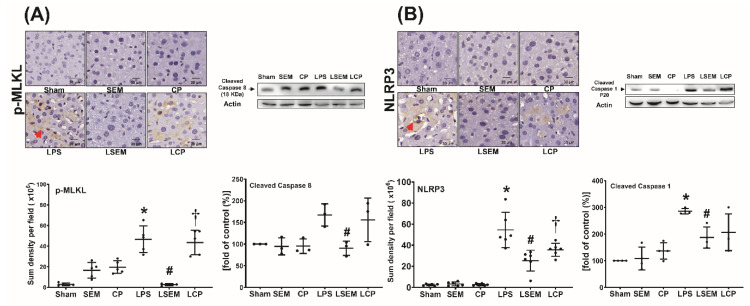
SEM18 peptide mitigates endotoxin-induced (**A**) necroptosis and (**B**) pyroptosis in the liver, as measured at 24 h after lipopolysaccharide (LPS) or normal saline (NS) administration. (**A**) Representative microscopic images of phosphorylated mixed lineage kinase domain-like pseudokinase (p-MLKL, red arrow) in liver tissues (200×) measured using immunohistochemistry assay and the quantitative sum intensity of p-MLKL. Representative gel photography of cleaved caspase 8 and actin (the internal standard) in liver tissues and the cleaved caspase 8/actin band density. (**B**) Representative microscopic images of nod-like receptor protein 3 (NLRP3, red arrow) in liver tissues assayed using immunohistochemistry staining assay (200×) and the quantitative sum intensity of NLRP3. Representative gel photography of cleaved caspase 1 and actin (the internal standard) in liver tissues evaluated using immunoblotting assay and the cleaved caspase 1/actin band density. Sham: the NS group; SEM: the NS plus SEM18 peptide group; CP: the NS plus control peptide (CP) group; LPS: the LPS (15 mg/kg) group; LSEM: the LPS (15 mg/kg) plus SEM18 peptide group; LCP: the LPS (15 mg/kg) plus CP group. Data are the mean ± standard deviation. Data of immunohistochemistry staining assay and immunoblotting assay were derived from five and three mice from each group, respectively. * *p* < 0.05, the LPS group vs. the sham group; ^#^ *p* < 0.05, the LSEM group vs. the LPS group; ^†^ *p* < 0.05 the LCP group vs. the LSEM group.

Analyses of necroptosis and pyroptosis in liver tissues measured at 24 h after LPS or NS administration are illustrated in Figure 5A,B. Expression levels of necroptosis-related protein (i.e., phosphorylated mixed lineage kinase domain-like pseudokinase (p-MLKL) and cleaved caspase 8) and pyroptosis-related protein (i.e., NLRP3 and cleaved caspase 1) in liver tissues of the sham, SEM, and CP groups were low. Expression levels of p-MLKL, NLRP3, and cleaved caspase 1 in the LPS group were significantly higher than those in the sham group (all *p* < 0.001). Although the expression level of cleaved caspase 8 in the LPS group was higher than that in the sham group, the between-group differences did not reach statistical significance. Compared with the LPS group, significantly lower expression levels of p-MLKL, cleaved caspase 8, NLRP3, and cleaved caspase 1 were observed in the LSEM group (*p* < 0.001, *p* = 0.035, *p* < 0.001, and *p* = 0.024, respectively). The differences between the LSEM and LCP groups paralleled those between the LSEM and LPS groups, except that the differences in cleaved caspase 8 and cleaved caspase 1 between the LSEM and LCP groups were nonsignificant.

### 2.7. SEM18 Peptide Mitigates LPS-Induced Apoptosis and Autophagy Upregulation in the Liver

**Figure 6:**
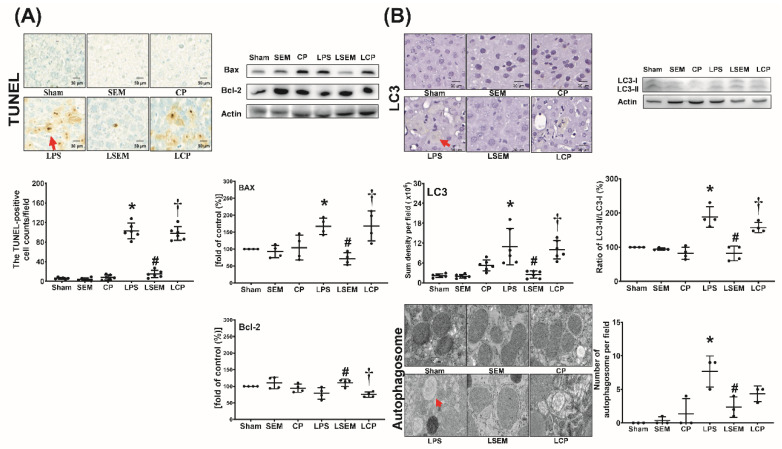
SEM18 peptide mitigates endotoxin-induced (**A**) apoptosis and (**B**) autophagy upregulation in the liver, as measured at 24 h after lipopolysaccharide (LPS) or normal saline (NS) administration. (**A**) Representative microscopic images of DNA fragmentation (red arrow) in liver tissues (200×) assayed using terminal deoxynucleotidyl transferase dUTP nick-end labeling (TUNEL) method and the mean TUNEL-positive cell counts (per 0.25 mm^2^). Representative gel photography of apoptosis-related proteins (i.e., the proapoptotic Bax and the antiapoptotic Bcl-2) and actin (the internal standard) in liver tissues assayed using immunoblotting assay and the band densities of Bax/actin and Bcl-2/actin. (**B**) Representative microscopic images of microtubule-associated protein 1A/1B-light chain 3 (LC3, red arrow) in liver tissues (200×) measured using immunohistochemistry assay and the quantitative sum intensity of LC3. Representative gel photography of LC3-I, LC3-II, and actin (the internal standard) in liver tissues and LC3-II/LC3-I ratio. Representative transmission electron microscopic images of autophagosome (red arrow) in liver tissues (6000×) and the mean autophagosome numbers (per 2 μm^2^). Sham: the NS group; SEM: the NS plus SEM18 peptide group; CP: the NS plus control peptide (CP) group; LPS: the lipopolysaccharide (15 mg/kg) group; LSEM: the LPS (15 mg/kg) plus SEM18 peptide group; LCP: the LPS (15 mg/kg) plus CP group. Data are the mean ± standard deviation. Data of immunohistochemistry staining assay, immunoblotting assay, and transmission electron microscopic were derived from five, four, and three mice from each group, respectively. * *p* < 0.05, the LPS group vs. the sham group; ^#^ *p* < 0.05, the LSEM group vs. the LPS group; ^†^ *p* < 0.05, the LCP group vs. the LSEM group.

Analyses of apoptosis and autophagy upregulation in liver tissues measured at 24 h after LPS or NS administration are illustrated in Figure 6A,B. The terminal deoxynucleotidyl transferase dUTP nick-end labeling (TUNEL)-positive cell counts, expression levels of pro-appoptotic Bax and autophagy-related protein microtubule-associated protein 1A/1B-light chain 3 (LC3), ratios of LC3-II/LC3-I, and mean numbers of autophagosomes in liver tissues of the sham, SEM, and CP groups were low. These parameters were significantly higher in the LPS group than in the sham group (*p* < 0.001, *p* = 0.036, *p* < 0.001, *p* < 0.001, and *p* < 0.001, respectively) and the LSEM group (*p* < 0.001, *p* < 0.001, *p* < 0.001, *p* < 0.001, and *p* = 0.013, respectively). The differences between the LSEM and LCP groups also paralleled those between the LSEM and LPS groups, except that the difference in autophagosome numbers between the LSEM and LCP groups was not significant. The antiapoptotic Bcl-2 expression levels in the sham, SEM, and CP groups were not significantly different (Figure 6A). Notably, the Bcl-2 expression levels in the LPS and sham groups were not significantly different, wheras the Bcl-2 expression level in the LSEM group was significantly higher than that in the LPS and LCP groups (*p* = 0.020 and 0.008, respectively).

### 2.8. Exogenous TNF-α Counteracts the Effects of SEM18 Peptide

Exogenous TNF-α (recombinant mouse TNF-α; R&D Systems, Minneapolis, MN, USA) was used to confirm whether the therapeutic effects of SEM18 peptide were mediated by decreasing TNF-α binding to TNFR1. A set of mice (*n* = 5, designated as the LSEM + TNF-α group) were treated with LPS as described earlier. Two hours after LPS, exogenous TNF-α (0.3 mg/kg i.p.) was administered, followed by SEM18 (0.3 mg/kg, i.p.). Supplemental doses of exogenous TNF-α administration (0.15 mg/kg, i.p.) and SEM18 (0.15 mg/kg, i.p.) were administered every 2 h three times. These mice were closely monitored for 24 h (starting from LPS administration). All mice survived the experiment. These mice were then euthanized for further investigations.

**Figure 7:**
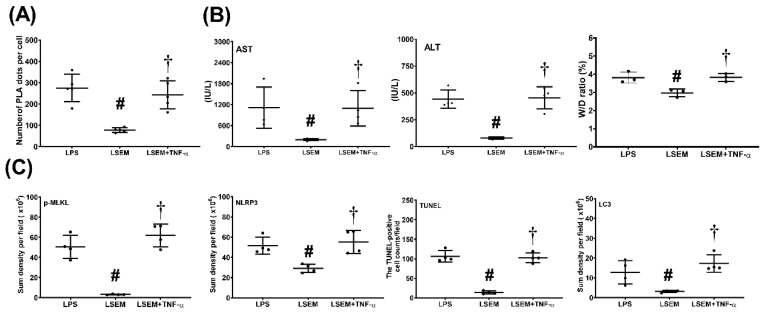
Exogenous tumor necrosis factor (TNF)-α counteracts the effects of SEM18 peptide. (**A**) Binding of TNF-α to TNF receptor 1, as measured at 24 h after lipopolysaccharide (LPS) administration using immunofluorescence staining of proximity ligation assay (PLA). (**B**) Plasma concentrations of aspartate aminotransferase (AST) and alanine aminotransferase (ALT) and the wet/dry (W/D) weight ratio of the liver tissues, as measured at 24 h after LPS administration. (**C**) The quantitative sum intensity of phosphorylated mixed lineage kinase domain-like pseudokinase (p-MLKL), nod-like receptor protein 3 (NLRP3), and microtubule-associated protein 1A/1B-light chain 3 (LC3) in liver tissues, as measured at 24 h after LPS administration using immunohistochemistry staining assay. For apoptosis analysis, liver tissues were assayed using the terminal deoxynucleotidyl transferase dUTP nick-end labeling (TUNEL) method and the mean TUNEL-positive cell in liver tissues (per 0.25 mm^2^) was calculated, as measured at 24 h after LPS administration. LPS: the LPS (15 mg/kg) group; LSEM: the LPS (15 mg/kg) plus SEM18 peptide group; LSEM + TNF-α: the LPS (15 mg/kg) plus SEM18 peptide and TNF-α group. Data are the mean ± standard deviation. Data of PLA, plasma ALT and AST, W/D weight ratio, immunohistochemistry staining assay, and TUNEL assay were derived from five, four, four, four, and four mice from each group, respectively. ^#^ *p* < 0.05, the LSEM group vs. the LPS group; ^†^ *p* < 0.05, the LSEM + TNF-α group vs. the LSEM group.

Figure 7 illustrates the effects of exogenous TNF-α. Our data demonstrated that hepatic PLA signal intensity (*p* < 0.001), plasma concentrations of AST and ALT (*p* = 0.003 and *p* = 0.014, respectively), and the wet/dry weight ratio (*p* = 0.001) of liver tissues were significantly higher in the LSEM + TNF-α group than in the LSEM group. Moreover, expression levels of p-MLKL, NLRP3, and LC3 in liver tissues and the TUNEL-positive cell counts were significantly higher in the LSEM + TNF-α group than in the LSEM group (*p* < 0.001, *p* < 0.001, *p* = 0.004, and *p* < 0.001, respectively). Of note, the differences between the LSEM and LSEM + TNF-α groups paralleled those between the LSEM and LPS groups. Collectively, these data illustrated the effects of exogenous TNF-α on counteracting the therapeutic effects of SEM18 peptide.

## 3. Discussion

This study confirmed that SEM18-based peptide therapy, when administered after sepsis induction, can decrease the systemic inflammatory response, improve survival rates, and mitigate liver injury in septic mice. These data provided clear evidence to confirm the therapeutic effects of SEM18 peptide against sepsis. As SEM18 peptide is a TNF-α-binding decoy, it can be an attractive alternative to the current TNF-α inhibitors and may serve as a novel therapeutic approach against sepsis.

Production of TNF-α from activated macrophages, as mentioned above, can be observed in the early phase of sepsis [2]. The essential role of this early surge of circulating TNF-α in initiating the development of a systemic inflammatory response during sepsis is confirmed by previous data that infusion of exogenous TNF-α into human or experimental animals can induce a systemic inflammatory response similar to the response observed during septic shock [22,23,24,25]. TNF-α production is mediated via the TLR/NF-κB pathway in activated macrophages during sepsis [1]. NF-κB is a crucial transcription factor that regulates maximal expression of a wide array of inflammatory mediators, including cytokines and chemokines [26,27]. Of note, TNF-α per se is a strong activator of NF-κB [1]. With its upregulation, TNF-α can in turn activate NF-κB and further enhance the production of inflammatory mediators. In line with this concept, we believe that SEM18 peptide can bind with circulating TNF-α to decrease NF-κB activation and subsequently mitigate inflammatory mediator upregulation. Although the present study did not investigate NF-κB expression, data from this study support this concept. Our data showed that the circulating concentrations of TNF-α and IL-1β, as well as hepatic concentrations of TNF-α and pro-IL-1β, in septic mice with SEM18 peptide therapy were significantly lower than in septic mice without SEM18 peptide therapy. These data also demonstrated that SEM18 peptide can decrease systemic inflammatory response and hepatic inflammation induced by sepsis. Moreover, it is established that the systemic inflammatory response induced by sepsis may damage vital organs and cause multiple organ failures in sepsis patients [2,3]. If uncontrolled, the systemic inflammatory response may eventually result in mortality in severe septic patients [22,23]. As SEM18 peptide can decrease systemic inflammatory response in septic mice, it is reasonable to observe from our data that SEM18 peptide therapy can improve survivorship in septic mice.

Moreover, data from this study demonstrated that the SEM18 peptide can serve as a TNF-α-binding decoy and decrease binding of TNF-α to TNFR1 in liver tissues induced by endotoxin. Moreover, this study confirmed our hypothesis that SEM18 peptide therapy can mitigate liver injury induced by endotoxin. Effects of SEM18 peptide therapy on inhibiting the crucial mechanisms in mediating endotoxin-induced liver injury, including inflammation, mitochondrial injury, necroptosis, pyroptosis, apoptosis, and autophagy upregulation, were also observed in this study. Of note, our data demonstrated that exogenous TNF-α can counteract the effects of SEM18 peptide on inhibiting TNF-α binding to TNFR1. Exogenous TNF-α can also offset the above described therapeutic effects of SEM18 peptide. Collectively, these data demonstrated the therapeutic potentials of SEM18 peptide against sepsis. Moreover, as SEM18 peptide was administered after sepsis induction, data from this study support the concept that SEM18 peptide possesses potent therapeutic potentials against sepsis. Data from this study, thus, have clinical significance and warrant further investigations.

It is established that activation of the TNF-α/TNFR1 pathway can cause pyroptosis, necroptosis, and apoptosis [4,5,6,7,8,9]. This concept is confirmed by this study, as our data demonstrated that SEM18 peptide can act by decreasing TNF-α binding to TNFR1 to mitigate pyroptosis, necroptosis, and apoptosis induced by endotoxin in liver tissues. Moreover, TNF-α can directly cause mitochondrial injury and dysfunction [10,11]. It is also established that dysfunctional mitochondria can induce apoptosis (i.e., the mitochondrial-dependent pathway) and trigger the processes of necroptosis and pyroptosis [10,11,12,13]. Data from this study also supported this concept, as our data demonstrated that SEM18 peptide can mitigate mitochondrial injury and inhibit apoptosis, necroptosis, and pyroptosis induced by endotoxin in mice. Autophagy, a process of self-degradation, is responsible for removing misfolded protein and damaged organelles (e.g., mitochondria) and, thus, is generally considered a survival mechanism [28]. However, dysregulated autophagy can also induce cell death [14,28]. Although existing data indicate that the TNF-α/TNFR1 pathway does not directly modulate activity and expression of autophagy, crosstalk between autophagy and various forms of cell death has been highlighted [14]. As this study was conducted to investigate the effects of SEM18 peptide therapy on modulating mitochondrial injury, necroptosis, pyroptosis, and apoptosis in septic mice, we also investigated autophagy expression in this regard. Our data revealed that endotoxin can upregulate autophagy expression in liver tissues, and SEM18 peptide therapy can mitigate the levels of autophagy upregulation in septic mice. Moreover, our data revealed that changes in autophagy upregulation correlate well with those in mitochondrial injury, necroptosis, pyroptosis, and apoptosis. Because the TNF-α/TNFR1 pathway does not directly modulate autophagy upregulation, as mentioned above, we believe that SEM18 peptide may very likely act through mitigating the levels of necroptosis, pyroptosis, apoptosis, and mitochondrial injury to indirectly decrease the levels of autophagy upregulation in septic mice.

**Figure 8:**
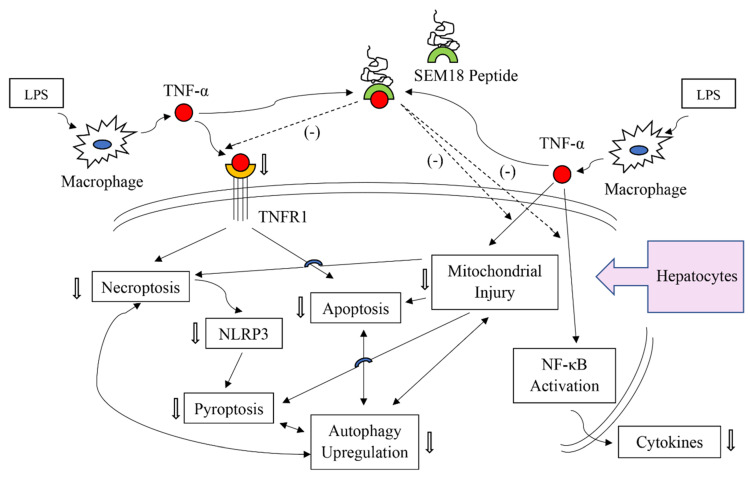
Illustration of the proposed possible mechanisms of SEM18 peptide on inhibiting endotoxin-induced liver injury in hepatocytes. LPS: lipopolysaccharide; TNF-α: tumor necrosis factor-α; TNFR1: tumor necrosis factor receptor 1; NF-κB: nuclear factor-κB. → indicating confirmed inhibition effects of SEM18 peptide; ⇢ indicating presumed effects of SEM18 peptide.

The present study confirmed the therapeutic effects of SEM18 peptide against liver injury induced by endotoxin. Hepatocytes (i.e., the functional cells of the liver) constitute approximate 80% of liver mass [29]. It is likely that the observed SEM18 peptide effects against endotoxin-induced liver injury were mainly from hepatocytes. This concept is supported by a follow-up cellular study conducted in our laboratory. Our data demonstrated that SEM18 peptide can mitigate cytokine (e.g., IL-1β) production induced by TNF-α in primary hepatocytes (please see Figure S1, Supplementary Materials). Taking these above-mentioned data and information together, we propose some mechanisms that underlie the therapeutic effects of SEM18 peptide against endotoxin-induced liver injury. We propose that SEM18 peptide can act by binding with circulating TNF-α to decrease hepatocyte exposure to circulating TNF-α. With decreased exposure to circulating TNF-α, SEM18 peptide can then inhibit activation of NF-κB, upregulation of cytokines, and injury of mitochondria in hepatocytes. Moreover, with decreased exposure to circulating TNF-α, SEM18 peptide can then mitigate TNF-α binding to the transmembrane death receptor TNFR1 and subsequently inhibit the death processes of necroptosis, pyroptosis, apoptosis, and autophagy upregulation in hepatocytes. The proposed possible mechanisms are summarized in Figure 8.

It is established that a dysfunctional inflammatory response is essential in mediating organ damage in sepsis [30]. Inflammatory cytokines (e.g., TNF-α, IL-1β, and IL-6) stimulate systematic inflammation, whereas anti-inflammatory cytokines (e.g., IL-1 receptor antagonist (IL-1Ra) and IL-10) inhibit inflammation and enhance healing [31,32]. Balance between inflammatory and anti-inflammatory cytokines constitutes a crucial component of inflammatory network balance [33,34]. Notably, sepsis induces robust production of inflammatory cytokines, which disrupts the balance in the inflammatory network and results in a dysfunctional inflammatory response [33,34]. Data from the present study confirmed that SEM18 peptide can inhibit upregulation of inflammatory cytokines. Although the present study did not investigate the expression of anti-inflammatory cytokines, we believe that SEM18 peptide may act through, at least in part, inhibiting inflammatory cytokines to help restoring inflammatory network balance. Similar to the inflammatory network, sepsis also induces an unbalanced cell-specific host response [35]. In addition to upregulation of cellular signaling, sepsis is also associated with downregulation of neutrophil action [35]. Acting through the process of phagocytosis and the release of reactive oxygen species and antimicrobial peptides, neutrophils constitute the first line of defense against pathogens [36]. After engulfing and killing invading pathogens, neutrophils then go through the process of apoptosis to prevent the release of histotoxic contents [36]. Neutrophil apoptosis, thus, plays an essential role for inflammation resolution [35]. However, neutrophil apoptosis can be inhibited by TNF-α [37]. In line with this notion, we conjecture that SEM18 peptide may act through binding with TNF-α to restore the function of neutrophils. Of note, recent data demonstrated impaired kinase activity in neutrophils of sepsis patients [38]. These data provide a possible explanation for the downregulation of neutrophil action observed in sepsis. This study did not investigate the effects of SEM18 peptide in this regard. However, judging from the beneficial effects of SEM18 peptide observed in this study, it is likely that SEM18 peptide may exert significant effects on restoring the kinase activity of neutrophils and helping to restore the function of neutrophils.

Similar to sepsis, the processes of TNF-α upregulation and its subsequent binding to TNFR1 also play crucial roles in immune-mediated diseases, e.g., autoimmune disease [39]. Clinical data have demonstrated the therapeutic effects of anti-TNF-α therapies against autoimmune diseases, including rheumatoid arthritis, juvenile rheumatoid arthritis, inflammatory bowel disease (IBD), ankylosing spondylitis, psoriasis, and psoriatic arthritis [39,40]. Moreover, the power of anti-TNF-α therapies for complicated patients (e.g., pregnant IBD patients) has also been highlighted [40]. For instance, on the basis of data from a systematic review of the literature, suggestions of continuing anti-TNF-α therapies throughout pregnancy in pregnant IBD patients and switching to anti-TNF-α therapies in pregnant IBD patients who have a disease flare with other therapies have been made [41]. Notably, anti-TNF-α therapies are immunosuppressive and, thus, may cause reactivation of latent infection, e.g., tuberculosis and hepatitis B [41]. Therefore, safety issues have been raised regarding the clinical application of anti-TNF-α therapies [41]. As SEM18 peptide can inhibit TNF-α upregulation and decrease TNF-α binding to TNFR1, we conjecture that SEM18 peptide possesses the potential to serve as an effective alternative to current anti-TNF-α therapies and may exert beneficial effects against those above-mentioned immune-mediated diseases. Future studies to elucidate the therapeutic effects and safety of SEM18 peptide in this regard are needed before further conclusions can be drawn.

Although this study confirmed the therapeutic potential of SEM18 peptide against sepsis, several crucial issues remain unelucidated. First, the pharmacodynamics of SEM18-based peptide therapy were not investigated. Only one therapeutic protocol was employed. Therefore, the question of whether the effects of SEM18 peptide are dose-dependent remains unstudied. The most effective therapeutic protocol also remains to be developed. Second, the peptide has a short in vivo half-life [42]. Data from this study confirmed this concept. To facilitate delivery of a sufficient amount of peptide to the target organ, the researchers of this study designed a therapeutic protocol of three supplemental doses after the initial dose of SEM18 peptide. Our data indicated that this protocol can exert significant effects against sepsis. However, to increase its clinical therapeutic efficacy, a certain modification (e.g., conjugation with polymer) that can efficiently lengthen the in vivo half-life of SEM18 peptide is still required. Third, a trend of lower IL-6 expression was observed in the LSEM group when compared to the LPS group in this study. However, the trend did not reach statistical significance. One possible explanation may be that these data were derived from a relatively small sample size. More studies are needed before we can conclude the definitive effects of SEM18 peptide in modulating IL-6 upregulation induced by endotoxin. Moreover, our data also revealed no significant differences in some assays between the LSEM and LCP groups (i.e., cleaved caspase 8, cleaved caspase 1, and autophagosome number). These data seemed to suggest that the control peptide might also exert certain effects against endotoxin. Fourth, we used a murine monomicrobial sepsis model to investigate the short-term effects of the SEM18 peptide. Future studies are required to definitively demonstrate the therapeutic effects of SEM18 peptide against sepsis. Lastly, studies indicated that LPS signaling and TNF-α may play pivotal roles in the host defense against common viruses [43,44]. For instance, LPS signaling may act through the NF-κB and Janus kinases/signal transducer and activator of transcription (i.e., JAK-STAT) signaling pathways to restrict murine norovirus replication [44]. Moreover, anti-TNF-α therapy (e.g., infliximab) may abrogate the effects of TNF-α against rotavirus [44]. In line with this notion, it is likely that SEM18 peptide may in turn promote vulnerability to common viruses. Again, more studies are needed before further conclusions can be drawn in this regard.

## 4. Materials and Methods

### 4.1. Animals and Study Approval

This study employed adult male Balb/cJ mice (8–10 weeks old, National Laboratory Animal Center, Taiwan). Mice were fed a standard laboratory chow, provided water ad libitum, and maintained in a 12 h light/12 h dark cycle. All animal studies were approved by the Institutional Animal Use and Care Committee of Taipei Medical University (LAC-2017-0206, approved on 19 September 2017). The care and handling of the mice complied with the guidelines of the US National Institutes of Health.

### 4.2. Peptide Design and Synthesis

SEM18 (molecular weight: 2161.54 Da) and a random peptide (i.e., the CP, molecular weight: 2161.54 Da) was designed using the “dock proteins protocol” (ZDOCK, BIOVIA Discovery Studio 3.5; BIOVIA, San Diego, CA, USA), as previously reported [21]. SEM18 peptide and CP were then synthesized (Mission Biotech, Taipei, Taiwan) for experiments. The purity values of SEM18 peptide preparation and CP preparation were both 95%, as tested using high-performance liquid chromatography.

### 4.3. Pharmacokinetic Analysis

A set of mice were used for pharmacokinetic analysis of SEM18 peptide. To facilitate investigation, SEM18 peptide was conjugated with biotin (Abcam, Cambridge, UK). Then, biotin-conjugated SEM18 peptide (0.3 mg/kg, i.p.) was administered. Mice were anesthetized with zoletil/xylazine (40/10 mg/kg, i.p.) and a midline laparotomy was performed to facilitate blood drawing via aortic puncture. Blood drawing was performed at 0 min, 5 min, 30 min, 1 h, 2 h, and 4 h after administration of SEM18 peptide. The plasma concentrations of SEM18 peptide were determined via measuring plasma biotin concentrations using ELISA.

### 4.4. Ex Vivo Bioluminescence Imaging

A set of mice were used for monitoring biodistribution of SEM18 peptide. To facilitate imaging, SEM18 peptide was conjugated with rhodamine B (Abcam). Then, rhodamine B-conjugated SEM18 peptide (0.3 mg/kg, i.p.) was administered. At 0 h and 2 h after SEM18 peptide administration, mice went through the processes of anesthesia, laparotomy, and blood drawing, as mentioned above, and the organs of heart, lung, liver, kidney, and spleen were harvested. A bioluminescence image assay was performed using an in vivo imaging system (IVIS Lumina XRMS; PerkinElmer, Waltham, MA, USA), and the images were then analyzed using the Living Image software (PerkinElmer).

### 4.5. Endotoxemia Mouse Model and Peptide Therapy

LPS (*E. coli* 0127:B8 endotoxin; Sigma-Aldrich, St. Louis, MO, USA), as mentioned above, was used to induce monomicrobial sepsis and liver injury [45]. Mice were randomly allocated to the sham, SEM, CP, LPS, LSEM, and LCP groups. As mentioned above, at 2 h after LPS or NS administration, the first dose of the peptide (SEM18 or CP, 0.3 mg/kg, i.p.) was administered, followed by three supplemental doses of the peptide (SEM18 or CP, 0.15 mg/kg, every 2 h, i.p.). The timing of administration of the peptides was decided on the basis of previous data from healthy volunteers showing that circulating TNF-α concentrations peaked at approximately 60–90 min after endotoxin [2]. Three supplemental doses were administered considering the short in vivo half-life of the peptides [42]. The first dose of SEM18 peptide was determined according to our preliminary data that 0.3 mg/kg was the lowest effective dose of SEM18 peptide that could mitigate increases in biomarkers of liver injury (i.e., plasma concentrations of AST and ALT) in mice, as measured at 6 h after LPS.

### 4.6. Survival Assay

A set of mice were used for the survival assay. Mice were treated as mentioned earlier and then closely monitored for 48 h (starting from LPS or NS administration) to determine the survival rate in each group. Death was recognized as the disappearance of blood pressure pulsation.

### 4.7. Liver Tissue Harvesting and Wet/Dry Weight Ratio Assay

Another set of mice were used for the remaining experiments. Mice were treated as mentioned earlier. At 24 h after LPS or NS, the surviving mice were anesthetized, as mentioned above. Mice were then euthanized through decapitation, and liver tissues were harvested. The right and median lobes of the liver were snap-frozen and stored at −80 °C for further analysis, and the left lobe was fixed with 10% formalin (Sigma-Aldrich) for histological analysis. The freshly harvested caudate lobe was weighed and placed in the oven at 80 °C for 24 h and then weighed again. The wet/dry weight ratio was then calculated [46].

### 4.8. Binding of TNF-α and TNFR1

Immunofluorescence staining of the PLA was performed to evaluate the binding of TNF-α to TNFR1 [47]. The assay was performed using a DuoLink Mouse Rabbit in situ PLA kit (Sigma-Aldrich) according to the manufacturer’s instructions. In brief, paraffin sections of liver tissues were incubated with primary antibodies of TNF-α and TNFR1 (both from Abcam), followed by oligonucleotide-labeled secondary antibodies (i.e., PLA probes). Staining with DAPI (Sigma-Aldrich) was also performed to detect nuclei. Sections were then imaged (DeltaVision Elite microscope; GE Healthcare, Marlborough, MA, USA) and analyzed (Image J; free program from https://imagej.nih.gov/ij/).

### 4.9. Biomarkers and Histological Analysis of Liver Injury

Plasma concentrations of AST and ALT were analyzed with an autoanalyzer (Vitros 750; Johnson & Johnson, New Brunswick, NJ, USA). In addition, the formalin-fixed liver tissues were embedded in paraffin wax, serially sectioned, and then stained with hematoxylin and eosin. Morphological characteristics (including PMN infiltration, interstitial edema, focal necrosis, and hemorrhage/congestion) were evaluated under a light microscope, and Suzuki scores (i.e., the sum of scoring of congestion (0: none; 4: severe), vacuolization (0: none; 4: severe), and necrosis (0: none; 4: >60%)) were calculated to determine liver injury levels [48,49].

### 4.10. ELISA

Plasma concentrations of TNF-α, IL-1β, and IL-6 were analyzed using ELISA. Hepatic concentrations of TNF-α, pro-IL-1β, and IL-6 were also analyzed using ELISA. After the plasma and tissue samples were processed, as reported previously [50], commercial ELISA kits (Enzo Life Science, Farmingdale, NY, USA) were employed, and ELISA was performed as per the manufacturer’s protocols.

### 4.11. Immunohistochemistry Staining Assay

The status of necroptosis, pyroptosis, and autophagy was evaluated using an immunohistochemistry staining assay, as previously reported [50]. Paraffin sections of liver tissues were incubated with the primary antibody of p-MLKL (Abcam) [51], NLRP3 (Abcam) [7], or LC3 (Cell Signaling, Danvers, USA) [52]. All sections were scanned (TissueGnostics Axio Observer Z1 microscope; TissueGnostics GmbH, Vienna, Austria) and analyzed (Image J).

### 4.12. TUNEL Assay

The key characteristic of apoptosis, that is, DNA fragmentation, in the liver tissues was assayed using the TUNEL method [20]. An in situ cell death detection kit (Roche, Indianapolis, IN, USA) was employed, and staining of the apoptotic cells in liver tissues was performed according to the manufacturer’s protocols. Staining with DAPI (Sigma-Aldrich) was also performed to detect total nuclei. All sections were scanned and analyzed, as described previously, and the mean TUNEL-positive cell count in each group was determined using five randomly selected fields (0.25 mm^2^).

### 4.13. Immunoblotting Assay

Snap-frozen liver tissues were processed as previously reported [48]. Equal amounts of proteins (65 μg) were then separated by electrophoresis and then transferred to nitrocellulose membranes (Bio-Rad Laboratories, Hercules, CA, USA). The membranes were incubated with primary antibody of necroptosis-related protein cleaved caspase 8 (Abcam) [51], pyroptosis-related protein cleaved caspase 1 (Abcam) [7], apoptosis-related proteins Bax and Bcl-2 (both from Abcam) [6], autophagy-related protein LC3 (LC3-I and LC3-II; Cell Signaling) [52], or actin (as the internal standard; Sigma-Aldrich). Bound antibody was detected through chemiluminescence (ECL Plus kit; Amersham, Buckinghamshire, UK). Protein band density was measured using densitometry (ImageJ). The LC3-II/LC3-1 ratio (i.e., an indicator of autophagosome formation) [52] was calculated.

### 4.14. Transmission Electron Microscopy Analysis

Mitochondrial injury (i.e., deformation of mitochondrial cristae or inner mitochondrial membrane disruption) and autophagosome formation (i.e., cytoplasmic components enclosed in double-membraned vesicles) were assayed using transmission electron microscopy analysis, as previously reported [10,53]. Harvested liver tissues were fixed, washed, and fixed again in 2% osmium tetroxide and embedded in Epon 812 resin (all from Sigma-Aldrich). The grids containing sections were stained with 2% uranyl acetate and 0.2% lead acetate (both from Sigma-Aldrich) followed by ultrathin sectioning (60–70 nm). The sections were examined with a transmission electron microscope (Hitachi HT-7700; Hitachi, Tokyo, Japan). Five random fields (2 μm^2^) were selected to count the numbers of intact mitochondria, as well as autophagosomes, in each group by a pathologist blinded to the treatment.

### 4.15. Statistical Analysis

Data were presented as the mean ± standard deviation. Between-group differences were analyzed using one-way analysis of variance and post hoc pairwise comparisons with Tukey’s test. Kaplan–Meier analysis was performed to analyze the between-group differences in 48 h survival rates. A *p*-value < 0.05 was considered statistically significant. SPSS v21.0 (SPSS, Somers, NY, USA) was used for statistical analysis.

## 5. Conclusions

The peptide-based TNF-α-binding decoy therapy using SEM18 can exert therapeutic effects and mitigate the adverse effects of endotoxin on experimental septic mice.

## Supplementary Materials

The following are available online at https://www.mdpi.com/1424-8247/13/10/280/s1: Figure S1. Effects of SEM18 peptide on mitigating interleukin-1β (IL-1β) upregulation in primary hepatocytes treated with tumor necrosis factor-α (TNF-α).
