# Supplementary material for: Peptide-Based TNF-α-Binding Decoy Therapy Mitigates Lipopolysaccharide-Induced Liver Injury in Mice
Source: Pharmaceuticals (Basel). 2020 Sep 29;13(10):280. doi: 10.3390/ph13100280 (PMC7600127; doi:10.3390/ph13100280)
Supplement: Supplementary file 1 [file pharmaceuticals-13-00280-s001.pdf]

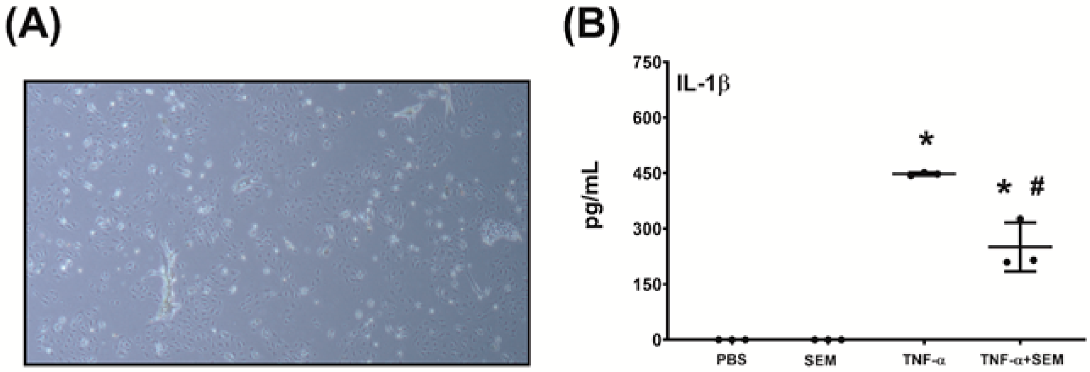

Figure S1. Effects of SEM18 peptide on mitigating interleukin-1β (IL-1β) upregulation in primary hepatocytes treated with tumor necrosis factor-*α* (TNF-*α*). (**A**) Morphology of primary hepatocytes, isolated from adult male Balb/cJ mice liver. Primary hepatocytes were observed with an inverted phase-contrast microscope (100×) (Carl Zeiss, Oberkochen, Germany). (**B**) Concentrations of IL-1β in freshly harvested culture media. Primary hepatocytes were treated with phosphate-buffered saline (PBS, Sigma-Aldrich), PBS plus SEM18 peptide (10 μg/mL), TNF-*α* (40 ng/mL, Sigma-Aldrich), or TNF-*α* (40 ng/mL) plus SEM18 peptide (10 μg/mL) and denoted as the PBS, SEM, TNF-*α*, and TNF-*α* + SEM groups, respectively (n = 3 in each group). In the TNF-*α* + SEM group, SEM18 peptide was added 2 h before TNF-*α*. At 6 h after PBS or TNF-*α*, cell cultures were harvested. IL-1β concentrations in freshly harvested culture media were measured using enzyme-linked immunosorbent assay. Data are the mean ± standard deviation. * $p < 0.05$ vs. the PBS group; # $p < 0.05$ vs. the TNF-*α* group.
